# Longitudinal Knowledge Assessment as a driver for learning: insights from a nationwide survey of endocrinologists

**DOI:** 10.1210/jendso/bvag176

**Published:** 2026-08-04

**Authors:** Bradley Michael Gray, Junhui Qian, Weifeng Weng, Benjamin Jacob Chesluk, Brendan Joel Barnhart, Rebecca Sue Lipner, Rozalina Grubina McCoy

**Affiliations:** Assessment and Research Department, American Board of Internal Medicine, Philadelphia, PA 19106, USA; Assessment and Research Department, American Board of Internal Medicine, Philadelphia, PA 19106, USA; Assessment and Research Department, American Board of Internal Medicine, Philadelphia, PA 19106, USA; Assessment and Research Department, American Board of Internal Medicine, Philadelphia, PA 19106, USA; Assessment and Research Department, American Board of Internal Medicine, Philadelphia, PA 19106, USA; Assessment and Research Department, American Board of Internal Medicine, Philadelphia, PA 19106, USA; Division of Endocrinology, Diabetes, & Nutrition, Department of Medicine, University of Maryland School of Medicine, Baltimore, MD 21201, USA; Institute for Health Computing, University of Maryland, North Bethesda, MD 20852, USA

**Keywords:** longitudinal knowledge assessment, maintenance of certification, continuing medical education, board certification, physician learning, clinical practice change

## Abstract

**Context:**

The American Board of Internal Medicine (ABIM) started offering the Longitudinal Knowledge Assessment (LKA) to endocrinologists as a 10-year Maintenance of Certification alternative in 2022. Participants answer 30 open-book online questions quarterly, receive real-time question-specific feedback and overall feedback via progress reports starting in year 2, and are assessed for competency every 5 years.

**Objective:**

To examine whether the LKA encouraged learning, motivated further study, and/or changed patient care.

**Design:**

We surveyed endocrinologists in 2024 who started LKA in 2022–2024 about their learning, study motivated by progress report feedback, patient care changes, and changes in the use of information resources. Results were weighted for nonresponse.

**Setting:**

Mid-career endocrinologists.

**Intervention:**

ABIM's LKA.

**Main outcomes:**

Reports of learning, motivated study, and patient care changes.

**Results:**

We surveyed 1649 endocrinologists, of whom 1015 responded to the survey (62% response). Overall, 95% of respondents reported learning while answering questions. Of those viewing their progress reports, 68% reported that they were motivated to study by the feedback. Motivated study was 17 percentage points greater for physicians with bottom vs top quartile first progress report preliminary scores. Additionally, 53% of physicians reported that LKA participation resulted in changes or planned changes to patient care. These reports were about 30 percentage points greater among physicians reporting that participation led to changes in the use of information resources, learning broader than question level, learning from question references, and study motivated by progress report feedback.

**Conclusion:**

Endocrinologists’ LKA participation resulted in self-reports of patient care changes, broad learning, and motivated study.

The American Board of Internal Medicine (ABIM) introduced the Endocrinology Longitudinal Knowledge Assessment (LKA) option for Maintenance of Certification (MOC) in 2022. This new approach to certification aims to combine assessing the competency of mid-career physicians with enhancing participating physicians’ knowledge, both directly (through immediate feedback while answering questions) and indirectly (through study motivated by periodic score reports) (1). LKA participants answer 30 questions online quarterly in an open-book format, are asked to rate question–answer confidence, receive real-time feedback after each question (including answers, rationales, and references), and are assessed in 5-year cycles. Quarterly progress reports, starting in the second year of participation, include preliminary scores, performance relative to other LKA takers, and performance both by content area (eg, diabetes, thyroid disease, and others) and answer confidence level (2). The goal of LKA is both to encourage learning to address identified gaps in knowledge and as a means to assess competency (1).

The LKA includes several evidence-based features that promote learning, including spaced learning (exposure to materials interspersed with other activities), repeated testing, interleaving of content (ie, mixing to-be-learned subjects rather than focusing on one subject at a time), and motivation in the form of meaningful consequences (3-7). Several studies have shown that performance on ABIM's traditional MOC exam is associated with better care quality and patient outcomes (8-15), and a recent study suggests that this association is also present for LKA performance in that Internal Medicine (IM) preliminary LKA scores were found to be associated with reduced mortality and readmission rates for hospitalized Medicare beneficiaries (16).

Recent survey studies of general internists participating in ABIM's IM LKA and pediatricians participating in the American Board of Pediatrics’ Maintenance of Certification–Peds longitudinal assessment (which is structured similarly to ABIM's LKA) found that physicians report real-time learning as the result of participating in these longitudinal assessments as well as further independent study motivated by progress report feedback and that participation led to changes or planned changes in patient care (17, 18). However, no study has yet examined the degree to which these findings generalize to subspecialties of internal medicine, such as Endocrinology, Diabetes, and Metabolism (Endocrinology), where the knowledge base is narrower, deeper, and more specialized. This is particularly pertinent to understanding the impact of LKA participation on clinical care decisions and self-directed learning practices, where subspecialists’ in-depth knowledge acquisition and application may differ from that of generalists. Keeping current with medical knowledge is especially important for endocrinologists, considering the recent rapid evolution of evidence-based practice across multiple endocrine disorders (19-26).

We therefore surveyed the first 3 cohorts of endocrinologists participating in ABIM's Endocrinology LKA about the degree to which LKA promoted learning and motivated further study, and whether participation resulted in perceived changes to patient care. Because LKA participants are allowed to use information resources that they may use in everyday practice (except another person), including UpToDate, artificial intelligence tools, and other online resources, we also investigated the degree to which participation led to reported changes in the use of these resources in day-to-day practice.

## Methods

**Figure 1:**
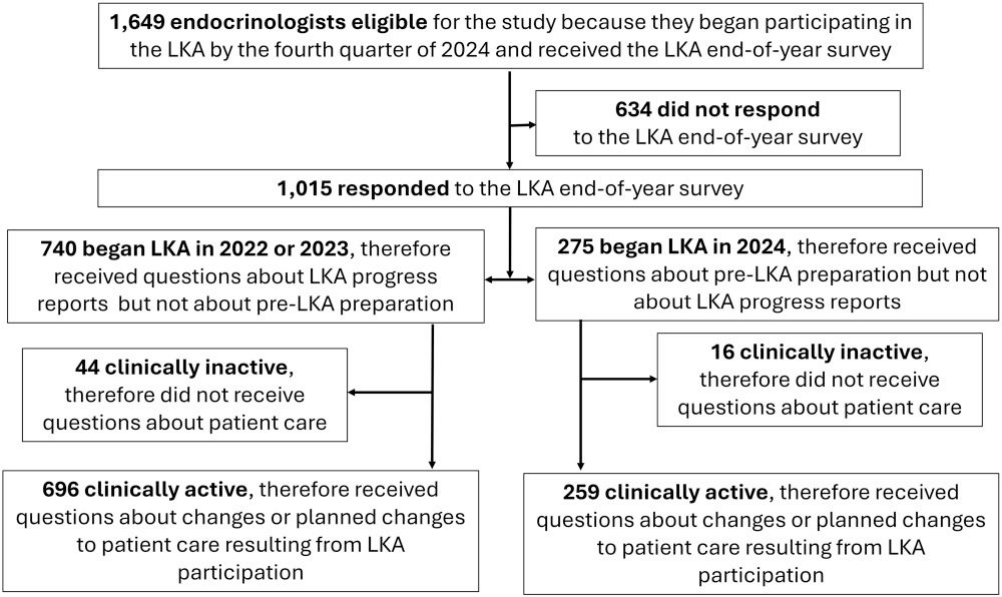
American Board of Internal Medicine Longitudinal Knowledge Assessment (LKA) endocrinologist participant study inclusion criteria.

The survey target population was physicians who enrolled in the Endocrinology LKA between September 2022 and June 2024. After these physicians completed their 30th question of the fourth quarter of 2024, a link to the survey was provided in the LKA platform. Therefore, the 2022 initial enrollment cohort would have just completed their third year of participation when they were asked to complete the survey, while the 2024 cohort would not have yet answered enough LKA questions to receive their first progress reports when surveyed (Fig. 1). The survey link was also emailed to participating physicians so they could, alternatively, complete the survey at a later date. Monthly reminders were sent to physicians who had received the survey but had not responded. A final reminder was sent to nonresponders in early January 2025, when the survey was also emailed to LKA participants who had not completed their fourth quarter questions and so had not previously received the link to the survey.

### Survey instrument

Mirroring the survey of IM LKA participants, survey questions covered the effects of LKA participation on learning, whether further study was motivated by progress report feedback, the effects on patient care, and pre-enrollment preparation [see Supplement section 1 (27)] (18). In addition to these topics, the new survey also included a question about whether participation in LKA had affected the use of information resources in day-to-day practice. The survey instrument was user-tested through a pilot test with 5 physicians enrolled in LKA.

Survey questions related to learning from immediate feedback when answering questions included learning from feedback in terms of getting a question correct or incorrect, learning from the answer rationale, and learning from the references for this rationale. Physicians who reported learning from any of these sources were then asked to choose whether the resulting scope of learning was narrowly related to specific LKA questions (referred to as question level) or was broader in scope. Physicians who enrolled in LKA in 2022 and 2023 and therefore had access to their first progress reports were asked whether each of the progress report components (ie, performance relative to all other LKA takers, performance by content area and confidence level, and performance compared with a preliminary passing score) motivated further study and whether the study was narrow (ie, question level) or broader in scope. Physicians who enrolled in LKA in 2024 were asked about pre-LKA preparation (ie, the time spent per week and the number of weeks/months spent preparing). We did not ask participants in other enrollment cohorts these questions to avoid recall bias resulting from the time difference between when they answered the survey and when their pre-LKA preparation might have occurred. Physicians who reported that they were clinically active were asked whether participation in LKA resulted in changes or planned changes to patient care. LKA participants were also asked whether participation affected how they use information resources in their day-to-day practice. To explore the degree to which other factors were related to reports of learning, motivated study, and changes to patient care, we merged performance data from the first progress report for the 2022 and 2023 LKA initial enrollment cohorts. We then constructed indicators for LKA score quartile based on 2024 scores for all Endocrinology LKA participants.

### Statistical analysis

Physicians participating in LKA may be incentivized to address potential knowledge gaps identified by feedback on questions and progress report feedback in terms of comparisons of their performance relative to other physicians, an assessment standard and performance by content area. We tested these hypotheses using logit regressions where the dependent measures were indicators for self-reports of learning being broader than question level and reports of further study motivated by progress report feedback types. In these regressions, the key explanatory variables were indicators of first progress report score quartile. Regressions also controlled for LKA enrollment year to account for time differences between responding to the survey and receiving the progress report, as well as participation duration.

To measure adjusted mean dependent measure outcomes by exam quartile, applying coefficient estimates from these logit regressions, we compared simulated probabilities of our measures of motivated study across the applicable physician sample assuming each physician's score was in the first, second, third, or fourth score quartile categories, holding all other characteristics constant (28). To test hypotheses of motivated study resulting from different types of LKA feedback, we then compared the adjusted probabilities across the fourth, third, and second quartiles compared with the first quartile.

Using the same approach, we also tested the hypothesis that patient care changes or planned changes were positively associated with other factors related to learning and motivated study including change in the use of information resources in day-to-day practice, learning broader than question level, learning from references (something that required additional effort on the part of physicians), study motivated by progress report feedback, and performance as measured by score quartile. To do this, we included these factors as explanatory variables in separate regressions (using binary indicators for each measure) where we also controlled for enrollment year. We limited these regressions to the applicable physicians (eg, for the regression that examined study motivation by progress report feedback, we excluded the 2024 cohort since these physicians were not asked questions related to the progress report).

We adjusted for possible survey nonresponse bias using propensity score weighting with poststratification. Subjects were assigned a poststratification weight after being divided into quintiles based on estimated propensity scores (29-31). The estimated propensity scores were computed from a logistic regression model containing American Board of Internal Medicine (ABIM) administrative data describing physician characteristics available for the invited population. The outcome variable in the logistic regression model used to construct propensity scoring weights was a binary variable indicating that a physician in the sample frame had or had not responded to the survey. Predictors for the model were based on ABIM administrative data and were observed for both survey respondents and nonrespondents and were selected based on previous research where an association with ABIM survey response behavior was observed. The demographic characteristics used as predictors were an indicator variable representing a pass on the first attempt of initial certification, age, sex, census region, medical school country, and LKA cohort. After applying these weights, estimates reflect the overall study target population.

Hypothesis tests were 2-tailed, with *P* < .05 considered statistically significant. The study was deemed exempt from review by the Advarra Institutional Review Board. This study follows the Strengthening the Reporting of Observational Studies in Epidemiology (STROBE) reporting guidelines (32). All analyses were performed using Stata software version 18 (StataCorp).

## Results

We identified 1649 endocrinologists enrolled in the Endocrinology LKA between 2022 and 2024, of whom 1015 responded to the survey (62% response rate). Physicians who responded to the survey were similar to nonrespondents in terms of characteristics observable in both populations, including years beyond initial certification (ie, duration in clinical practice), sex, and progress report score (Table 1). Absolute standardized differences ≥0.10 were considered to indicate meaningful imbalance (33).

**Table 1:** Demographics of the sample frame vs the unweighted survey responders

Characteristic	Sampling frame (n = 1649)	Unweighted responders (n = 1015)	Nonresponders (n = 634)	Absolute standardized difference between unweighted responders and nonresponders
Female, %	58.9	58.3	59.6	0.02
Initial IM exam performance, %				
Passed first attempt	92.3	92.5	92.0	0.02
Census region, %				
West	20.4	19.1	22.6	0.08
Midwest	18.3	18.6	17.8	0.02
South	34.3	35.5	32.3	0.07
Northeast	24.1	23.8	24.5	0.01
Canada/other	2.9	3.0	2.8	0.01
Age, %				
<41	2.8	2.7	3.0	0.02
41-50	48.6	45.4	53.6	0.16
51-60	31.0	33.1	27.6	0.12
61+	17.7	18.8	15.8	0.08
US medical school graduate, %	57.5	59.0	55.1	0.08
Enrollment year, %				
2022	36.3	35.9	36.9	0.02
2023	36.1	37.0	34.5	0.05
2024	27.7	27.1	28.6	0.03

Standardized differences were calculated comparing unweighted respondents with nonresponders as the difference in proportions divided by the pooled SD.

Abbreviation: IM, internal medicine.

An exception to this was age, where we observed a small difference between responders and non-responders for the age categories 41 to 50 years and 51 to 60 years (absolute standardized difference of ≤0.16). Notably, after propensity-score weighting, we did not find significant observed characteristic differences between the sampled population and the responding population (absolute standardized differences of ≤0.01) (Table 2).

**Table 2:** Demographics of the sample frame vs the weighted survey responders

Characteristic	Sampling frame (n = 1649)	Weighted survey responders (n = 1015)	Absolute standardized difference
Female, %	58.9	58.9	<0.01
Initial IM exam performance, %			
Passed first attempt	92.3	92.5	0.01
Census region, %			
West	20.4	20.2	0.01
Midwest	18.3	18.5	<0.01
South	34.3	34.4	<0.01
Northeast	24.1	24.0	<0.01
Canada/other	2.9	2.9	<0.01
Age, %			
<41	2.8	2.8	<0.01
41-50	48.6	48.9	0.01
51-60	31.0	30.7	0.01
61+	17.7	17.6	<0.01
US medical school graduate, %	57.5	58.0	0.01
Enrollment year, %			
2022	36.3	36.4	<0.01
2023	36.1	36.3	<0.01
2024	27.7	27.3	0.01

Standardized differences were calculated by comparing weighted respondent estimates to the corresponding proportions in the sampling frame, with the sampling frame treated as the reference group.

Abbreviation: IM, internal medicine.

Of the 1015 respondents, 696 had access to a score report and therefore responded to related survey questions; 259 enrolled in 2024 and therefore were asked survey questions related to preenrollment preparation (see Fig. 1 for sample selection).

### Sample characteristics

Of responding physicians, 41.3% (SE = 0.013) initially certified after 2011 and 23.0% (SE = 0.012) before 2002; 58.9% (SE = 0.015) were women, 41.8% (SE = 0.015) were trained outside the United States, and 94.3% (SE = 0.007) reported being clinically active (see Table 1). The 2024 cohort was smaller than the 2022 or 2023 cohorts: 364 in the 2022 cohort, 376 in the 2023 cohort, and 275 in the 2024 cohort.

### Pre-LKA preparation

In total, 13.2% (SE = 0.0191) of the 2024 cohort reported that they prepared ahead of time for LKA, with 75.0% (SE = 0.067) and 44.9% (SE = 0.078) indicating that they prepared for 1 month and ≥3 months, respectively, before starting LKA.

### Learning from answering questions

**Figure 2:**
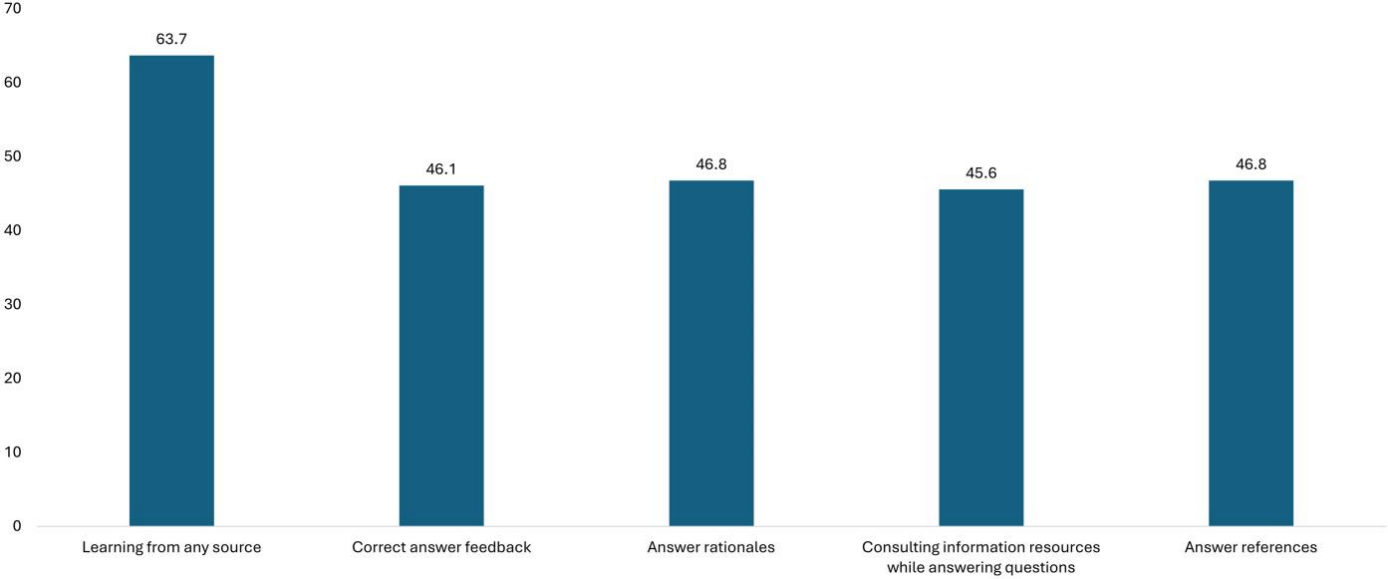
Percentage of endocrinologists participating in the American Board of Internal Medicine Longitudinal Knowledge Assessment (LKA) reporting that most learning resulting from participation was broader in scope than just related to specific LKA questions by learning source. Adjusted for enrollment year.

**Figure 3:**
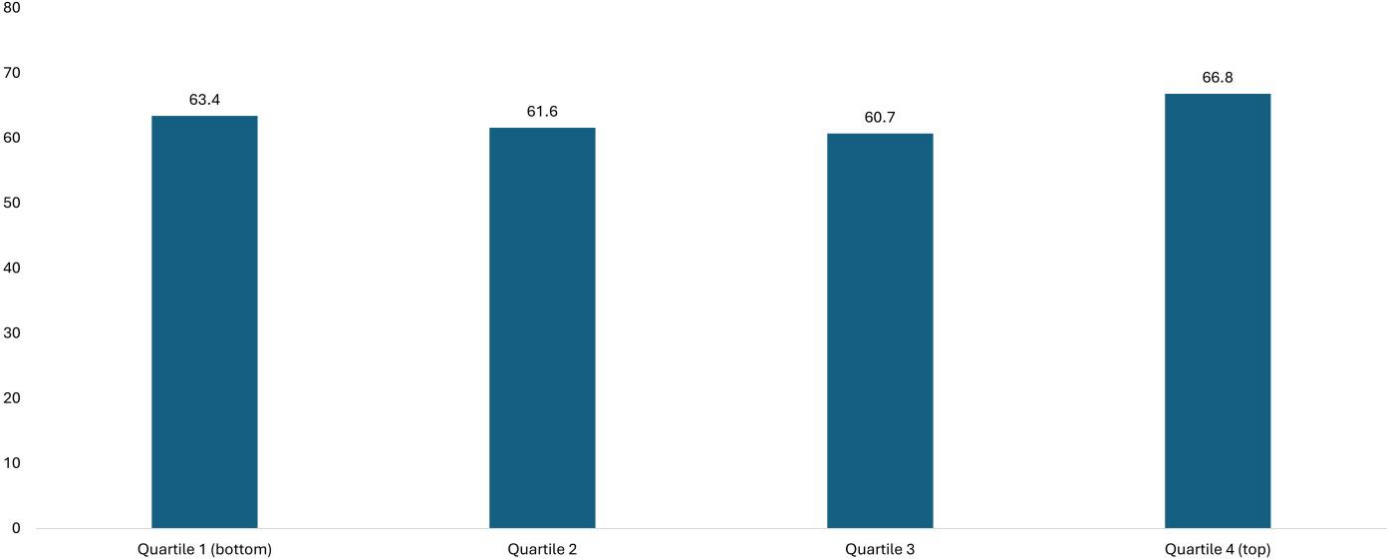
Percentage of endocrinologists participating in the American Board of Internal Medicine Longitudinal Knowledge Assessment (LKA) reporting that most learning while answering questions was broader in scope than question level by first-year LKA score quartile. Quartile differences are adjusted for enrollment year. The difference between first and second score quartiles is 1.8 (*P* = .676; 95% CI, −6.7 to 10.3). The difference between the first and third quartiles is 2.7 (*P* = .561; 95% CI, −6.4 to 11.9). The difference between the first and fourth quartiles is −3.4 (*P* = .433; 95% CI, −11.9 to 4.1).

Nearly all (94.5%; SE = 0.007), physicians reported that they learned while answering LKA questions either from correct/incorrect feedback (92.04%; SE = 0.009), question rationales (90.1%; SE = 0.010), consulting information resources (87.4%; SE = 0.011), or question references (64.3%; SE = 0.015). This learning was reported to be broader than just specific questions for just under 50% for each of these categories, and 63.7% (SE = 0.015) for at least 1 category (Fig. 2). We did not observe statistically significant regression-adjusted differences in reports of broad learning across physician score quartiles (Fig. 3).

### Use of information resources

Twenty-eight percent (SE = 0.015) of physicians reported that LKA participation had affected their use of information resources in their day-to-day practices.

### Progress report feedback and motivated study

**Figure 4:**
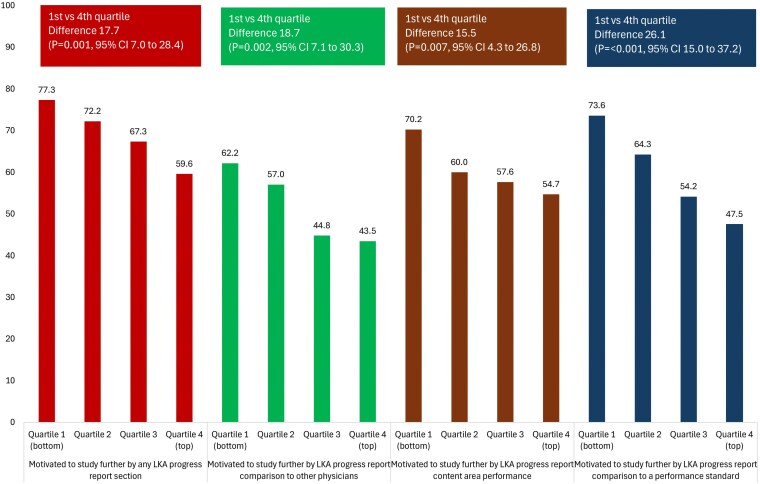
Percentage of endocrinologists participating in the American Board of Internal Medicine Longitudinal Knowledge Assessment (LKA) reporting being motivated to study further for each progress report feedback type (differentiated by color) by first year LKA score quartile. Quartile differences are adjusted for enrollment year and limited to 2022 and 2023 LKA initial enrollment year.

Of the 2022 and 2023 cohorts reported reviewing their progress reports, 88.2% (SE = 0.103) and 76.5% (SE = 0.018) of those who viewed the progress report found that it was useful for understanding their LKA performance, respectively. For those who viewed their progress report, 67.4% (SE = 0.020) indicated being motivated to study further by at least 1 of its feedback components. This percentage was 51.9% (SE = 0.021) for comparison to other physicians’ performance, 60.5% (SE = 0.020) for content area and confidence level feedback, and 59.7% (SE = 0.021) for progress score relative to the preliminary passing score. Among physicians who reported that progress reports motivated further study, 56.6% (SE = 0.025) reported that the scope of their study was broader than just the question level. This broader scope of study percentage was 52.4% (SE = 0.027), 55.8% (SE = 0.029) for feedback related to score comparisons with other physicians, 52.5% (SE = 0.027) for feedback by content area and confidence level, and 52.4% (SE = 0.027) for comparison with the preliminary passing score. We found that regression-adjusted reports of study motivated by progress report feedback were significantly more likely among physicians who scored in the bottom vs top quartile on their first-year LKA progress report (Fig. 4). For example, after controlling for enrollment year, the regression-adjusted percentage of physicians with bottom quartile scores who reported being motivated to study further was 77.3% (SE = 0.036) for physicians with bottom quartile scores vs 59.6% (SE = 0.04) for physicians with top quartile scores, corresponding to an absolute difference of 17.7 percentage points (*P* = .001; 95% confidence interval [CI] 7.0–28.4) and a nearly 30% difference comparing the top to the bottom quartile. We observed similar differences across each component of the progress report feedback.

### Changes or planned changes in patient care

**Figure 5:**
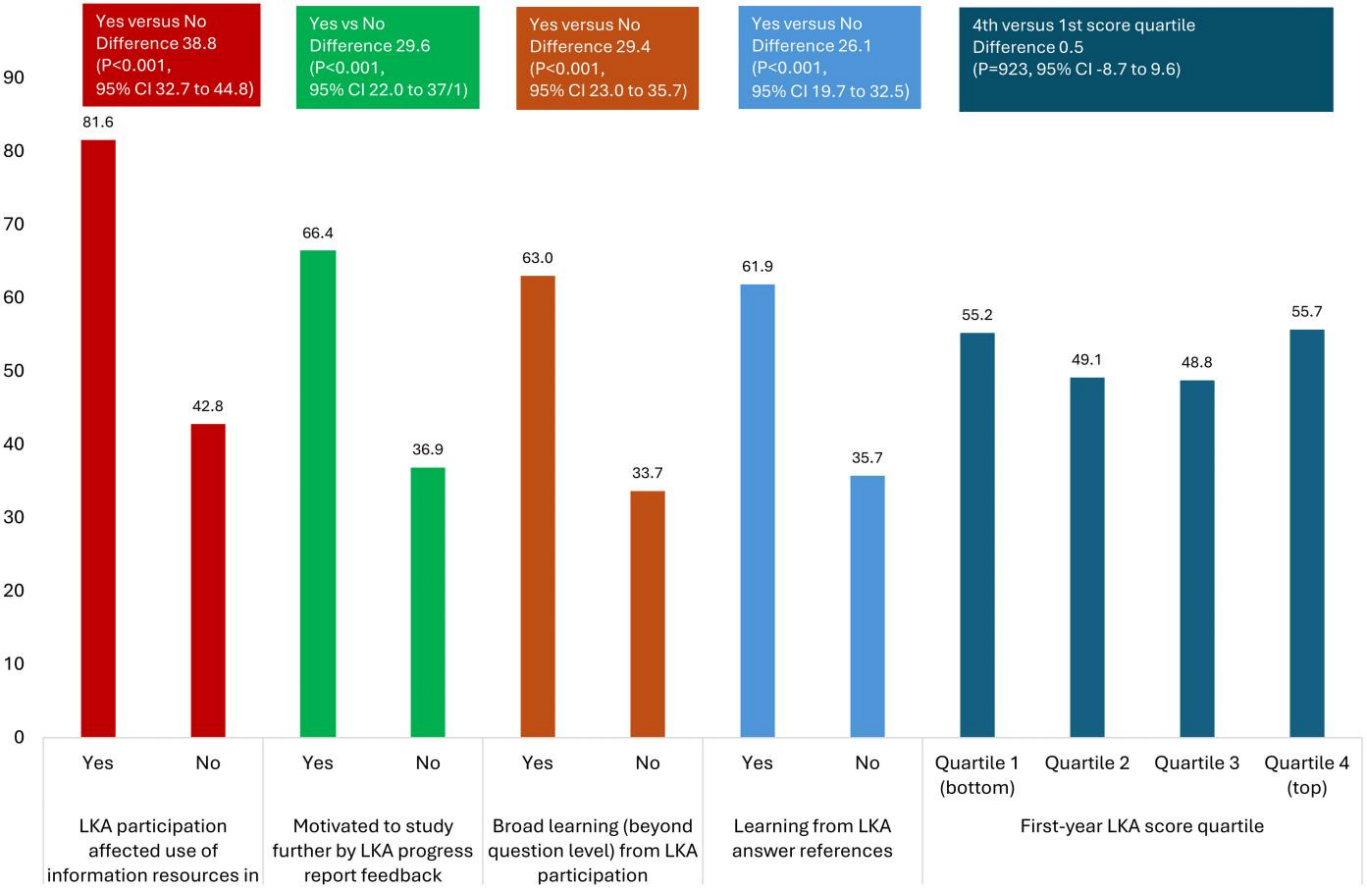
Percentage of clinically active endocrinologists participating in the American Board of Internal Medicine Longitudinal Knowledge Assessment (LKA) reporting that participation led to changes or planned changes to patient care by category (differentiated by color). Differences across subcategories are adjusted for enrollment year.

Over half (53.6%; SE = 0.0164) of physicians reported that they made or planned to make changes to patient care because of LKA participation. Specifically, 46.7% (SE = 0.015) reported that they had already changed their patient care, and 44.2% (SE = 0.016) reported that they planned to make changes in patient care. After controlling for enrollment year, we found that physician reports of changes or planned changes to patient care were significantly more likely for those who also reported that participation resulted in changed use of information resources, learning more broadly than question level, learning from references, and study motivated by progress report feedback (Fig. 5). The magnitudes of these associations were large; for example, the adjusted mean percentage reporting changes or planned changes to patient care resulting from participation was 81.6% (SE = 0.024) for physicians who reported that LKA had affected their use of information resources in everyday practice, as opposed to 42.8% (SE = 0.019) for those who did not report this, a difference of 38.8 percentage points (*P* < .001; 95% CI 32.7–44.8). This difference was close to 30 percentage points (each) for reports of broad learning, learning from references, and further study motivated by progress report feedback. Notably, we did not observe significant differences in reports of changes or planned changes to patient care across first score report quartiles.

## Discussion

Endocrinologists participating in ABIM's Endocrinology LKA reported that this participation supported their learning and motivated further study, especially for physicians who had relatively low scores on their first-year progress report. Importantly, their learning was generally broader in scope than just related to specific questions being asked, underscoring the potential of the LKA in improving knowledge and care quality more broadly. Indeed, a majority of physicians report that LKA participation either impacted their patient care already or they expect it to do so in the future. As such, participation in LKA appears to achieve its goal of supporting self-directed learning, improving knowledge, and enhancing care delivery beyond the assessment objective.

Given the rapid evolution of evidence-based practice across multiple endocrine disorders, this study is important in understanding ways to facilitate self-directed learning. The past decade, which corresponds to the interval elapsed since initial certification for LKA-eligible physicians included in this study, has witnessed paradigm shifts in several areas of endocrinology that fundamentally altered clinical decision-making, underscoring the importance of helping practicing endocrinologists stay abreast of innovations in care delivery and clinical guidance. In diabetes care, there has been a transformative shift from glucose-centric to complications-centric management with the emergence of sodium-glucose cotransporter-2 inhibitors and glucagon-like peptide-1 receptor agonists as foundational therapies based on cardiovascular and kidney outcome trials, shifting away from metformin as a standard first-line treatment for type 2 diabetes (19). Type 1 diabetes management has been revolutionized by rapid innovation in technology and the introduction of teplizumab, a first-in-class treatment to delay progression to clinical (stage 3) disease (20). Thyroid disease management has evolved toward more nuanced risk stratification of thyroid nodules, with guidelines emphasizing molecular testing and active surveillance to reduce overdiagnosis and overtreatment of low-risk thyroid cancers. The field has increasingly recognized the prevalence and clinical impact of primary aldosteronism, requiring systematic screening protocols and broad treatment (21-23). The advent of cancer immunotherapies has introduced a spectrum of immune-related endocrinopathies affecting the thyroid, pituitary, and adrenal glands that were rarely encountered in practice a decade ago (24). Recognition of subclinical Cushing syndrome in patients with adrenal incidentalomas has evolved, with growing evidence linking autonomous cortisol secretion to adverse metabolic and cardiovascular outcomes (25). Additionally, therapeutic options for osteoporosis have expanded significantly with novel anabolic agents like romosozumab, requiring endocrinologists to master new treatment algorithms (26). Our findings suggest that LKA's continuous learning model may be particularly well-suited to subspecialty practice where knowledge depth and currency in rapidly changing areas are essential for optimal patient care. The high reported rates of learning from LKA participation (95% of respondents) and patient care changes (54% of clinically active physicians) indicate that this assessment format effectively promotes ongoing knowledge acquisition and clinical application. That said, it is unclear how well learning reported by these physicians aligns with these changes in endocrinology clinical knowledge.

There are several potential explanations for the reported impact of LKA participation on patient care decisions. We found that physicians who noted their practice to be potentially or already changed as the result of LKA participation were primarily those who also reported that their learning was broad (vs single question) in scope, who reported learning from answer references, and for whom LKA participation affected their use of information resources in day-to-day practice. This suggests that physicians may use LKA-provided references as an opportunity to engage in self-directed learning and to identify or better use new sources of clinical information that can be helpful to them in the long term. Although we observed that lower-scoring physicians were significantly more likely to report that progress report feedback led to increased study, we did not observe associations between these scores and reports of patient care change. This might indicate that there needs to be better study guidance given to these physicians such as more personalized study plans and access to resources that aid in meaningful learning.

Our results corroborate the prior research, which focused on the IM LKA, finding that nearly all outpatient general internists participating in the IM LKA reported increased learning and motivation for further study, particularly with low first progress report scores (16). The rates of learning and motivated study in our endocrinology sample were comparable to those observed in general internal medicine, suggesting that the LKA format is effective across different scopes of practice. However, we observed somewhat lower rates of reported patient care changes among endocrinologists (54%) compared with general internists (approximately 75% in the IM LKA study). This difference may reflect the more specialized nature of endocrinology practice, where subspecialists may already maintain currency through focused continuing medical education (CME), specialty conferences, and specialty journals. Alternatively, general internists participating in IM LKA may be purposefully using the LKA assessment to maintain knowledge across a broader scope of practice, including areas outside their daily clinical focus, thus leading to more frequent practice changes. Similar to our findings, a previous qualitative study found that general internists’ preparation for the traditional IM MOC exam led both to reports of broad learning and changes to patient care. Especially in terms of broad learning, the similarity in findings is encouraging and suggests that, even though LKA is focused on learning while answering questions, the broad learning observed as the result of preparation for the traditional MOC exam was largely maintained. In terms of learning and effects on patient care, similar findings to ours were also reported for the ABP Maintenance of Certification-Peds longitudinal assessment (17). Thus, in summary, our study suggests that the benefits of LKA extend beyond generalist physicians to subspecialty areas of internal medicine.

This is also the first study, to our knowledge, to examine the effects of participation in longitudinal knowledge assessment on physicians’ use of information resources in clinical practices. Notably, we found that Endocrinology LKA participation led to reported changes in the use of information resources for about 30% of participants, and this change was further associated with a greater likelihood of also changing or planning to change patient care practices. This suggests that the LKA open-resource format may have resulted in an otherwise unexplored benefit of LKA, the encouragement of efficient and effective use of information resources that, in turn, may have positive impacts on patient care. This finding may be particularly relevant in the current era, in which artificial intelligence tools and clinical decision support systems are increasingly integrated into practice (17). Yet, research is needed to understand how LKA participation influences physicians’ approach to seeking and utilizing clinical information in practice and affects that use, as well as how use of information resources during LKA participation affects the validity of LKA assessment.

Results of our survey can inform the continued innovation in MOC that not only ensures accurate competency assessment but directly supports learning, evidence-based practice, and ultimately improved patient care outcomes. For example, actionable guidance and communication targeted at low performers and collaboration with educational partners, such as professional societies and continuing medical education providers, may support specific educational opportunities for low performers aimed at filling identified knowledge gaps. Considering the identified benefits on better self-reported use of information services, ABIM might consider providing guidance regarding how to use these resources. Additionally, ABIM may want to consider the benefits of this use while participating in LKA against the potential cost in terms of assessment integrity (eg, the risk that open-resource format could allow physicians to perform well without truly learning the material). However, more research is needed to understand whether the self-reported changes in learning and information use translate to improved care delivery and patient health outcomes.

An alternative way to incentivize lifelong learning is through state medical licensure CME requirements. While CME requirements were initially designed to ensure self-directed learning and competency in practice, it is unclear if they are able to fulfill these goals. CME requirements support participation in learning activities, but do not ensure their quality or comprehensiveness, cannot measure that comprehensive competency was achieved, and add undue burden on physicians. There is also substantial variation across states in CME requirements (eg, New York State has no CME requirement), and they disproportionately impact physicians practicing in low-resource settings without clear evidence of benefit (34-36). Research synthesizing self-assessment studies in various fields, including medicine, shows that choices of self-assessment study tend to be biased and do not enhance learning and retention. Past research also indicates that, more generally, physicians’ ability to self-assess is limited, especially for poor performers (35, 36). These biases stem from what we know about learners; they habitually review material in the order it was originally learned or stop studying too soon instead of focusing on areas of deficiency (37-41). That said, past research indicates that state licensure requirements encouraging CME were associated with higher scores on ABIM's traditional MOC exam (34). Although there is evidence that well-designed CME can have a lasting effect on a physician's medical knowledge, the overall evidence concerning CME effectiveness is mixed and many studies evaluating CME are low quality (17, 42-45). Considering LKA's assessment function and that past research indicates that physicians may need help in choosing CME activities, ideally, feedback in the form of LKA continuous assessment would act synergistically with CME by guiding physicians in terms of educational interventions that address identified gaps in knowledge.

### Strengths and limitations

This study has several important strengths. First, we achieved a response rate of 62%, which is substantially higher than typical physician survey response rates and reduces the potential for nonresponse bias. We also accounted for nonresponse through propensity weighting, and, even without propensity score weighting, we found no material differences between respondents and nonrespondents in key characteristics including years beyond initial certification, sex, and progress report scores, suggesting that our results are representative of the broader population of endocrinologists participating in LKA. An exception to this was age, where we observed a small difference between survey responders and non-responders. Second, this is the first study to examine the educational impact of the LKA in a medical subspecialty, evaluating whether the benefits of this assessment format extend to subspecialists with deeper, more focused clinical knowledge. Our findings demonstrate that LKA is effective in promoting learning and practice change even in a specialized field, supporting its potential applicability across multiple subspecialties. Third, we examined a comprehensive set of outcomes including not only learning and knowledge acquisition but also motivated study behaviors, changes in information resource utilization, and reported patient care changes. This multidimensional assessment provides a more complete picture of how LKA participation influences physician behavior and practice patterns. Fourth, our study design allowed us to examine differences across multiple cohorts of participants (2022, 2023, and 2024 enrollments), providing insights into both early experiences with LKA and patterns that persist over time. The inclusion of pre-LKA preparation data from the most recent cohort adds valuable information about how physicians approach this new assessment format.

Our study also has limitations. Survey response bias might be present. That said, we accounted for nonresponse bias empirically. Furthermore, the characteristics of respondents and nonrespondents were similar, except for age, where we observed a small difference. Additionally, the survey response rate meets the standard of leading biomedical research journals such as *JAMA* and *JAMA Internal Medicine* (≥60%, as shown in the Survey Study in Instructions for Authors [see Supplement section 2 (27)]. We cannot verify whether the self-reported learning or practice changes actually occurred or were substantive. Considering this and the cross-sectional nature of our study, our findings should not be viewed as providing evidence of the causal effect of LKA participation but rather the perception of these effects by participants. Our results may not generalize to other subspecialties of internal medicine. That said, the results of this study were similar to the results of survey-based studies for general internists and pediatricians. Our study focused on potential benefits of LKA participation and not the cost in terms of time and fees of this participation. Furthermore, future research is needed to examine the extent of learning, in particular as it relates to recent changes in knowledge and guidelines related to the care of patients treated by endocrinologists, the degree to which this learning was relevant to patient care, and the impact of LKA-supported learning on care quality and patient outcomes, the criteria validity of the assessment, a comparison to other educational opportunities as well as an examination of the cost of participation. Research is also needed to both compare the effectiveness of LKA with CME and other educational activities, as well as to measure the degree to which LKA helps guide physicians in terms of their choice of activities to address gaps in knowledge identified by LKA feedback.

## Conclusion

Participants in ABIM's Endocrinology LKA report learning from answering questions; the study was motivated by progress report feedback, especially for low-performing physicians, and for over half of participants, changes or planned changes to patient care. More research is needed to determine the degree to which reports of patient changes due to LKA participation reflect real measurable improvements in care delivery and patient health outcomes.

## Data Availability

The data for this study are confidential, but de-identified data can be obtained through an American Board of Internal Medicine data use agreement process.
